# Impact of systemic therapy after stereotactic radiosurgery in patients with limited brain-only metastasis

**DOI:** 10.1093/noajnl/vdaf214

**Published:** 2025-11-20

**Authors:** Jamiluddin J Qazi, Amanda E D Van Swearingen, David J Carpenter, Gloria Broadwater, Jim X Leng, Muzamil Arshad, Steven J Chmura, Rani Bansal, Laura Alder, Peter E Fecci, John P Kirkpatrick, Joseph K Salama, Trey C Mullikin, Scott R Floyd, Carey K Anders

**Affiliations:** Department of Radiation Oncology, Duke University Medical ­Center, Durham, North Carolina; Duke Center for Brain and Spine Metastasis, Duke Cancer Institute, Durham, North Carolina; Division of Medical Oncology, Duke Cancer Institute, Duke University Medical Center, Durham, North Carolina; Department of Radiation Oncology, Duke University Medical ­Center, Durham, North Carolina; Department of ­Radiation Oncology, Wellstar Paulding Hospital, Hiram, Georgia; Biostatistics Shared Resource, Duke Cancer Institute, Duke University Medical Center, Durham, North Carolina; Department of Radiation Oncology, Duke University Medical ­Center, Durham, North Carolina; Department of Radiation and Cellular Oncology, University of Chicago Medical Center, Chicago, Illinois; Department of Radiation and Cellular Oncology, University of Chicago Medical Center, Chicago, Illinois; Duke Center for Brain and Spine Metastasis, Duke Cancer Institute, Durham, North Carolina; Division of Medical Oncology, Duke Cancer Institute, Duke University Medical Center, Durham, North Carolina; Duke Center for Brain and Spine Metastasis, Duke Cancer Institute, Durham, North Carolina; Division of Medical Oncology, Duke Cancer Institute, Duke University Medical Center, Durham, North Carolina; Duke Center for Brain and Spine Metastasis, Duke Cancer Institute, Durham, North Carolina; Department of Neurosurgery, Duke University Medical Center, Durham, North Carolina; Department of Radiation Oncology, Duke University Medical ­Center, Durham, North Carolina; Duke Center for Brain and Spine Metastasis, Duke Cancer Institute, Durham, North Carolina; Department of Radiation Oncology, Duke University Medical ­Center, Durham, North Carolina; Radiation Oncology Clinical Service, Durham VA Health Care System, Durham, North Carolina; Department of Radiation Oncology, Duke University Medical ­Center, Durham, North Carolina; Duke Center for Brain and Spine Metastasis, Duke Cancer Institute, Durham, North Carolina; Department of Radiation Oncology, Duke University Medical ­Center, Durham, North Carolina; Duke Center for Brain and Spine Metastasis, Duke Cancer Institute, Durham, North Carolina; Duke Center for Brain and Spine Metastasis, Duke Cancer Institute, Durham, North Carolina; Division of Medical Oncology, Duke Cancer Institute, Duke University Medical Center, Durham, North Carolina

**Keywords:** brain metastases | SRS | systemic therapy | timing

## Abstract

**Background:**

The impact of systemic therapy (ST) on outcomes for patients with brain-only metastases (BrM) in the absence of extracranial disease (ECD) is not well established. We compared outcomes between patients with BrM treated with stereotactic radiosurgery (SRS) who received ST ≤3 months (mos), >3 mos, or not at all after SRS.

**Methods:**

We identified BrM patients who completed SRS across two institutions from 1/2015 to 12/2020. Intracranial progression after SRS was determined by brain MRI radiographic recurrence. Overall survival (OS) and intracranial progression free survival (iPFS) estimates were also generated.

**Results:**

In total, 342 patients with BrM were identified. Primary sites included lung (73%), breast (12%), and additional sites (15%). Almost half, 169 (49%), received no ST, 80 (23%) received ST ≤3 mos, and 93 (27%) received ST >3 mos after SRS. Median age was younger in the ST >3 mos cohort (60.5years) compared with ST <3mo (67.7years) and no ST (67.0years), *P* = .0002. Median OS differed significantly between groups: ST ≤3 mos with 24.9mos (95%CI: 16.6-51.1), ST >3 mos with 27.5mos (95% CI: 20.6-37.5), and no ST with 11.0mos (95%CI: 9-17.5), *P* = .002. Median iPFS differed significantly between groups: ST ≤3 mos 16.1mos (95% CI: 9.5-33.7), ST >3 mos 8.9mos (95% CI: 6.9-13.5), and no ST 10.0mos (95% CI: 6.7- 15.1). However, timing of ST was not significant multivariate analysis.

**Conclusions:**

In our cohort of BrM patients, ST after SRS improves OS regardless of timing. ST ≤3 mos may improve iPFS compared with ST >3 mos after SRS, which warrants further investigation. Appropriate patients with BrM should be referred for a multi-disciplinary discussion of ST following SRS.

Key Points

- Systemic therapy after SRS improves overall survival
- Early systemic therapy after SRS may improve local control

Importance of the Study

Many patients with brain metastases receive systemic therapy (ST) after stereotactic radiosurgery (SRS). It is not known whether the timing of ST initiation after SRS affects patient outcomes. This study investigates the effect of ST timing after SRS on outcomes in patients with metastatic disease limited to the brain.

Brain metastases (BrM) affect up to 30% of all cancer patients and are clinically challenging to treat.^1^^,^^2^ Initial management often involves stereotactic radiosurgery (SRS), either as definitive treatment or as an adjuvant therapy after resection. There are rates of local control after SRS exceeding 85%, and it is recommended in published guidelines.^3^^,^^4^ However, more than half of the patients subsequently have intracranial progression (ICP) distant to the treated site.^5–7^

To that end, many patients with metastatic cancer are also managed with systemic therapy (ST). Historically, chemotherapy was often the standard of care in this setting. However, in recent years, the advent of novel ST, such as immunotherapies and targeted therapies, have altered the treatment paradigms for this patient population.^1^^,^^8^ These novel therapies can have better bioavailability in the brain and are often used in the treatment of BrM.^1^^,^^8^

There is no consensus on exactly how this novel ST should be integrated into the care of patients with BrM treated with SRS.^9^ Typically, SRS (or resection and then SRS) is delivered initially, followed by the ST. However, there is minimal guidance for clinicians on exactly when the ST should be started and exactly what benefit it may provide. This question is even more relevant in the absence of extracranial disease (ECD); after definitive treatment of the known metastatic disease with SRS, when to initiate ST that may result in toxicity is not well characterized. In addition, the influence of the timing of ST after SRS on potential adverse effects, like radionecrosis, is not known.

In this retrospective study of patients with BrM treated with SRS and no ECD, we attempt to clarify the impact of the timing of ST initiation after SRS on patient outcomes.

## Methods

This retrospective study was approved and exempted by the Duke University Medical Center Institutional Review Board (Pro00113702). An informed consent waiver was granted due to the use of deidentified patient data from an approved database (Pro00108434).

We identified patients completing a first course of SRS across two institutions, Duke University Medical Center and the University of Chicago, from January 1, 2015, to December 31, 2020. Demographic, clinical, and treatment data was captured manually using chart review. Patients with active metastatic ECD were excluded; previously treated and stable ECD was allowed. Single or multi-fraction SRS cases were included. Patients were allowed to receive prior local therapy, including prior resection or whole brain radiation therapy (WBRT). Patients were also allowed to have received prior ST.

Patients were classified into the three groups of inte­rest: patients who began ST within 3 months (mos) of completion of the first course of SRS, patients who began systemic more than 3 mos after SRS, and patients who received no ST after SRS at all. ST included medications from the classes of chemotherapy, immunotherapy, and targeted therapy and must have been initiated for the purpose of treating metastatic oncologic disease. The primary objective was the outcome of overall survival (OS). Secondary objectives included intracranial progression free survival (iPFS), extracranial progression free survival (ePFS), predictors of short and long-term survival, comparing ICP between each ST group within each primary tumor cohort, and comparing the incidence of radiation necrosis between each ST group within each primary tumor cohort. ICP was defined as concern for progression based on multidisciplinary consensus using available clinical information, including radiology imaging and reports, as well as pathology if available. All time to event outcomes were defined from completion of first course of SRS. Short-term survival was defined as ≤2 years; long-term ­survival was defined as 2 years or greater.

### Statistical Analysis

Patient characteristics were summarized using median and range or mean and standard deviation for continuous variables, and categorical descriptors were summarized with frequencies and percentages. Kaplan–Meier methods were used to estimate OS, iPFS, and ePFS survival. OS was evaluated from the date of SRS (with a 3-mo landmark) to death and censored at last follow-up visit. iPFS was evaluated from the date of SRS completion to intracranial progressive disease and was censored at date of last follow-up or death. ePFS was evaluated from the date of SRS completion to extracranial progressive disease and was censored at date of last follow-up or death. Curves were compared using log-rank tests. Cox proportional hazards models were used to compare survival (OS, iPFS, and ePFS) among the timing of ST groups after adjusting for covariates, listed in Table 1. A multivariate logistic regression model with stepwise selection with selected level of entry 0.25 and level of stay 0.10 was used to predict short versus long term survival as well as intracranial progression free survival. Stepwise selection proceeded if ST timing was significant in the initial model. Within the three main histology groups, the proportions of patients with (a) distant disease control, (b) intracranial disease control, and (c) incidence of radiation therapy necrosis in the three treatment timing groups were compared using chi-square tests. Analyses were conducted using SAS software (Version 9.4; SAS Institute Inc., Cary, NC) and plots were created in the R language and environment for statistical computing (R Foundation for Statistical Computing, Vienna, Austria).

**Table 1.** Demographic and treatment details of each patient cohort

	Timing of systemic therapy				
	Sys therapy within 3 mo of local (*N* = 80)	Sys therapy more 3 mo after local (*N* = 93)	No sys therapy (*N* = 169)	Total (*N* = 342)	*P* value
Age at SRS					.0002^a^
Mean (SD)	65.6 (12.08)	59.8 (11.88)	65.6 (11.10)	64.0 (11.81)	
Median (Range)	67.73 (25.96, 86.92)	60.45 (29.56, 88.89)	66.97 (35.34, 88.02)	65.32 (25.96, 88.89)	
Karnofsky performance status, *n* (%)					.1798^b^
100	11 (13.8)	5 (5.4)	14 (8.3)	30 (8.8)	
90	35 (43.8)	39 (41.9)	63 (37.3)	137 (40.1)	
80	25 (31.3)	31 (33.3)	48 (28.4)	104 (30.4)	
70	7 (8.8)	10 (10.8)	24 (14.2)	41 (12.0)	
60	1 (1.3)	6 (6.5)	9 (5.3)	16 (4.7)	
50 or less	1 (1.3)	2 (2.2)	11 (6.5)	14 (4.1)	
Karnofsky performance status, *n* (%)					.2004^b^
<=80	34 (42.5)	49 (52.7)	92 (54.4)	175 (51.2)	
90-100	46 (57.5)	44 (47.3)	77 (45.6)	167 (48.8)	
Gender, *n* (%)					.1710^b^
Female	39 (48.8)	55 (59.1)	80 (47.3)	174 (50.9)	
Male	41 (51.3)	38 (40.9)	89 (52.7)	168 (49.1)	
Race category, *n* (%)					.0701^b^
White	67 (83.8)	60 (64.5)	118 (69.8)	245 (71.6)	
Black	11 (13.8)	26 (28.0)	39 (23.1)	76 (22.2)	
Other/unknown	2 (2.5)	7 (7.5)	12 (7.1)	21 (6.1)	
Primary site category, *n* (%)					.0293^b^
Breast	3 (3.8)	16 (17.2)	21 (12.4)	40 (11.7)	
Lung	69 (86.3)	61 (65.6)	118 (69.8)	248 (72.5)	
Skin/melanoma	1 (1.3)	7 (7.5)	4 (2.4)	12 (3.5)	
GI	1 (1.3)	2 (2.2)	10 (5.9)	13 (3.8)	
Gyn	2 (2.5)	1 (1.1)	2 (1.2)	5 (1.5)	
GU	1 (1.3)	3 (3.2)	10 (5.9)	14 (4.1)	
Unknown/other	1 (1.3)	2 (2.2)	3 (1.8)	6 (1.8)	
Head and neck	2 (2.5)	1 (1.1)	1 (0.6)	4 (1.2)	
Primary site limited to breast, lung, and melanoma, *n* (%)					.0049^b^
Breast	3 (4.1)	16 (19.0)	21 (14.7)	40 (13.3)	
Lung	69 (94.5)	61 (72.6)	118 (82.5)	248 (82.7)	
Skin/melanoma	1 (1.4)	7 (8.3)	4 (2.8)	12 (4.0)	
Missing	7	9	26	42	
Metastatic burden, *n* (%)					.7274^b^
<=5 metastases	72 (90.0)	85 (91.4)	157 (92.9)	314 (91.8)	
5+ metastases	8 (10.0)	8 (8.6)	12 (7.1)	28 (8.2)	
Systemic therapy received prior to SRS, *n* (%)					<.0001^b^
No	68 (85.0)	50 (53.8)	49 (29.2)	167 (49.0)	
Yes	12 (15.0)	43 (46.2)	119 (70.8)	174 (51.0)	
Missing	0	0	1	1	
Number of lines any systemic therapy received prior to SRS, *n* (%)					<.0001^b^
0	68 (85.0)	50 (53.8)	50 (29.6)	168 (49.1)	
1	6 (7.5)	24 (25.8)	83 (49.1)	113 (33.0)	
2-3	6 (7.5)	16 (17.2)	28 (16.6)	50 (14.6)	
4+	0 (0.0)	3 (3.2)	8 (4.7)	11 (3.2)	
Intervals between primary diagnosis and initial brain metastasis diagnosis, *n* (%)					<.0001^b^
<30 days between primary and brain met diagnoses	58 (72.5)	46 (49.5)	42 (24.9)	146 (42.7)	
30 days to 1 year between	12 (15.0)	14 (15.1)	42 (24.9)	68 (19.9)	
>1 to 2 years between	4 (5.0)	16 (17.2)	31 (18.3)	51 (14.9)	
2+ years between	6 (7.5)	17 (18.3)	54 (32.0)	77 (22.5)	
Interval from brain metastasis diagnosis to initial SRS or resection, *n* (%)					.0003^b^
within 21 days	64 (80.0)	75 (80.6)	105 (62.1)	244 (71.3)	
22-60 days	14 (17.5)	13 (14.0)	33 (19.5)	60 (17.5)	
>60 days	2 (2.5)	5 (5.4)	31 (18.3)	38 (11.1)	
Prior resection of brain metastasis, *n* (%)					.0017^b^
No	54 (67.5)	38 (40.9)	96 (56.8)	188 (55.0)	
Yes	26 (32.5)	55 (59.1)	73 (43.2)	154 (45.0)	
Prior whole brain radiation therapy, *n* (%)					.0001^b^
No	78 (97.5)	88 (94.6)	138 (81.7)	304 (88.9)	
Yes	2 (2.5)	5 (5.4)	31 (18.3)	38 (11.1)	
Any intracranial progression post-SRS, *n* (%)					.0207^b^
No	35 (43.8)	25 (26.9)	73 (43.2)	133 (38.9)	
Yes	45 (56.3)	68 (73.1)	96 (56.8)	209 (61.1)	
Symptomatic intracranial progression post-SRS, *n* (%)					.0385^b^
No	72 (90.0)	75 (80.6)	129 (76.3)	276 (80.7)	
Yes	8 (10.0)	18 (19.4)	40 (23.7)	66 (19.3)	
Radionecrosis post SRS, *n* (%)					.8299^b^
No	36 (80.0)	52 (76.5)	77 (80.2)	165 (78.9)	
Yes	9 (20.0)	16 (23.5)	19 (19.8)	44 (21.1)	
Missing	35	25	73	133	
Extracranial progression post-SRS, *n* (%)					<.0001^b^
No	36 (45.0)	32 (34.4)	112 (66.3)	180 (52.6)	
Yes	44 (55.0)	61 (65.6)	57 (33.7)	162 (47.4)	
ST post-intracranial progression post-SRS, *n* (%)					<.0001^b^
No	0 (0.0)	0 (0.0)	128 (75.7)	128 (37.4)	
Yes	80 (100.0)	93 (100.0)	41 (24.3)	214 (62.6)	
Number lines of chemotherapy post SRS					<.0001^a^
Mean (SD)	0.9 (0.84)	0.8 (0.90)	0.1 (0.35)	0.5 (0.76)	
Median (Range)	1.00 (0.00, 3.00)	1.00 (0.00, 5.00)	0.00 (0.00, 2.00)	0.00 (0.00, 5.00)	
Number of lines of immunotherapy post SRS					<.0001^a^
Mean (SD)	0.8 (0.62)	0.7 (0.63)	0.1 (0.36)	0.4 (0.59)	
Median (Range)	1.00 (0.00, 2.00)	1.00 (0.00, 2.00)	0.00 (0.00, 2.00)	0.00 (0.00, 2.00)	
Number of lines of targeted therapy post SRS					.0013^a^
Mean (SD)	0.4 (0.89)	0.5 (0.96)	0.1 (0.45)	0.3 (0.74)	
Median (Range)	0.00 (0.00, 6.00)	0.00 (0.00, 5.00)	0.00 (0.00, 3.00)	0.00 (0.00, 6.00)	

a Kruskal-Wallis *P* value.

b Chi- Square *P* value.

## Results

In this study, we identified 342 patients receiving an initial SRS course with BrM and without ECD; 169 patients (49.4%) did not receive any ST after SRS in the follow up period (Table 1); 80 patients (23.3%) received ST within 3mo of SRS; 93 patients (27.2%) received ST more than 3mo after SRS. Median age was younger in the ST >3 mos cohort (60.5years) compared with ST <3mo (67.7years) and no ST (67.0years), *P* = .0002. Karnofsky Performance Status (KPS) was 90 or greater in 48.8% of patients and did not significantly differ between groups, and 50.9% of patients were female. The most common primary site was lung (72.5%), followed by breast (11.7%), genitourinary (4.1%), gastrointestinal (4.1%), skin/melanoma (3.5%), unknown/other (1.8%), gynecologic (1.5%), and head and neck (1.2%). The distribution of primary sites differed significantly between groups, with lung primary patients comprising 86.3% (*n* = 69) of the ST <3mo cohort, 65.6% (*n* = 61) of the ST >3mo cohort, and 69.8% (*n* = 118) of the no further ST cohort.

Of the total patients, 91.8% (*n* = 314) had 5 or fewer BrM, which did not differ significantly between groups; 85% (*n* = 68) of patients in the ST <3mo cohort did not receive ST prior to SRS, compared with 53.8% (*n* = 50) of the ST >3mo, and 29.2% (*n* = 49) of the never receiving ST post-SRS groups, *P* < .001; 45% (*n* = 154) of the entire cohort had resection prior to SRS, which differed significantly between groups; 59.1% of the ST >3mo cohort had resection versus 32.5% and 43.2% of the ST <3mo and no ST post-SRS, respectively (*P* = .0017); 11.1% of the entire cohort had WBRT prior to SRS, with 18.3% of the no ST post-SRS cohort receiving WBRT compared with 2.5% and 5.4% of the ST <3mo and ST >3mo cohorts, respectively (*P* = .0207). See additional demographics in Table 1.

**Figure 1.**
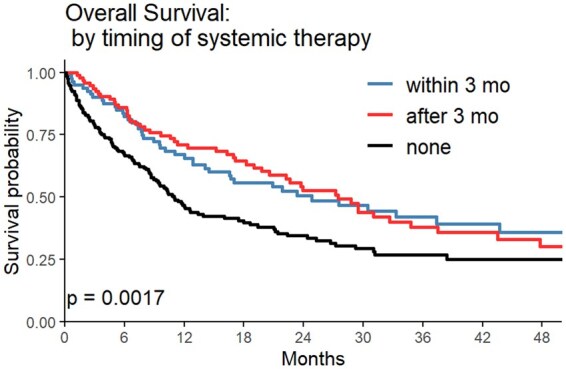
Overall survival Kaplan–Meier curve comparing systemic therapy (ST) within 3 mo of SRS, ST >3mo after stereotactic radiosurgery (SRS), and no ST after SRS.

**Figure 2.**
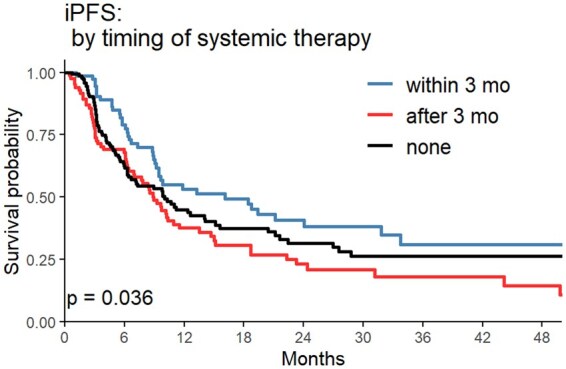
Intracranial progression free survival (iPFS) Kaplan–Meier curve comparing systemic therapy (ST) within 3mo from stereotactic radiosurgery (SRS), ST >3mo after SRS, and no ST after SRS.

**Figure 3.**
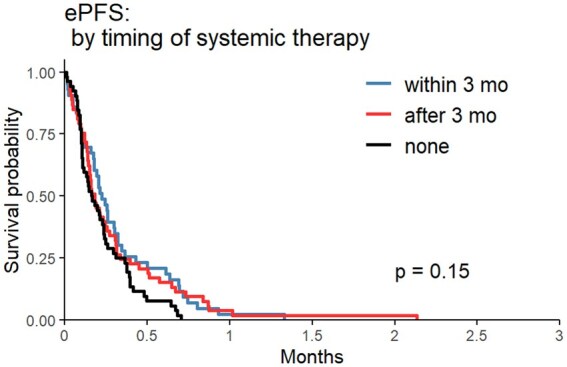
Extracranial progression free survival (ePFS) Kaplan–Meier curve comparing systemic therapy (ST) within 3mo from stereotactic radiosurgery (SRS), ST >3mo after SRS, and no ST after SRS.

Median OS was 25.85mo (95% CI: 16.55-51.05) in the ST <3mo cohort, 27.48mo (95% CI: 20.58-37.48) in the ST >3mo cohort, and 11.01mo (95% CI: 8.91-17.49) in the no ST post-SRS cohort (*P* = .0017) (Figure 1). In a stepwise multivariate model using candidate predictors of age, KPS, number of BrM, prior ST, tumor histology, and timing of ST, the ST < 3mo cohort had increased odds of long-term survival versus the no ST post-SRS cohort (OR = 2.178, 95% CI: 1.189-3.988, *P* = .016) (Table 2). Median iPFS was 16.09mo (95% CI: 9.46-33.72) in the ST <3mo cohort, 8.91mo (95% CI: 6.93-13.51) in the ST >3mo cohort, and 10.03mo (95% CI: 6.66-15.10) in the no ST post-SRS cohort (*P* = .036) (Figure 2). In a multivariate model adjusted for age, KPS, metastatic disease burden, primary sites, and prior ST, timing of ST was not a significant predictor of time to ICP post-SRS (*P* = .13, Table 3). Median ePFS did not differ significantly between groups in univariate analysis (Figure 3).

**Table 2.** Predictors of short versus long term survival

Predictors of short (<2 year) versus long (≥2 year) survival				
Predictors	Odds ratio	95% confidence limits		*P* value
No systemic therapy versus systemic therapy within 3 mo of SRS	2.178	1.189	3.988	.0156
Systemic therapy more 3 mo after SRS versus systemic therapy within 3 mo of SRS	1.089	0.566	2.095	
Age at SRS	1.017	0.995	1.039	.1215

Short term survival defined as less than 2 years; long term survival defined as 2 years or greater.

**Table 3.** Multivariate Cox regression model evaluating predictors of intracranial progression free survival (iPFS)

Covariate		*P* value	Hazard ratio	95% hazard ratio confidence limits	
Timing of systemic therapy	No systemic therapy after SRS	.1304	1.086	0.701	1.681
	Systemic therapy more 3 mo after SRS		1.445	0.947	2.206
Age at SRS		.3760	0.994	0.980	1.008
Karnofsky performance status	<=80	.3074	1.168	0.867	1.575
Metastatic burden	5 or more metastases	.0839	1.563	0.942	2.592
Primary site	Breast	0399	0.355	0.159	0.790
Primary site	Lung		0.485	0.245	0.960
Prior systemic therapy	No	.0313	0.675	0.472	0.965

In a multivariate model adjusted for age, KPS, metastatic disease burden, primary sites, and prior ST, timing of ST was not a significant predictor of ePFS post-SRS (*P* = .063, Supplementary Table 4). A Chi-square test comparing ECP within the breast primary patient cohort showed no difference between groups (*P* = .17, Supplementary Table 5). A Chi-square test comparing ECP in the lung primary patient cohort showed the patients with no ST post-SRS had lower rates of ECP (39.0%) compared with the ST <3mo cohort (62.3%) or the ST >3mo cohort (63.9%) (*P* < .001, Supplementary Table 6). There were too few patients to compare ECP within the melanoma/skin cohort.

A Chi-square test comparing ICP within the breast primary patient cohort showed that 33.33% (*n* = 1) of the ST <3mo cohort versus 93.75% (*n* = 15) of the ST > 3mo cohort had ICP (*P* = .007). A Chi-square test comparing ICP within the lung primary patient cohort did not show any significant differences. There were too few patients to compare ICP within the melanoma/skin cohort.

Neither of the Chi-Square tests comparing radiation necrosis between ST groups in the breast primary cohort nor the lung primary cohort was significant. There were too few patients to compare radiation necrosis within the melanoma/skin cohort.

## Discussion

In this retrospective multi-institutional cohort study of 342 patients with BrM, we show that starting ST after SRS, regardless of timing, is associated with improved survival. We show that initiating ST within 3mo of SRS was associated with increased intracranial control in univariate, but not multivariate, analysis. Additionally, within breast primary patients, we show that ST within 3mo of SRS was associated with increased intracranial control.

Prior work has shown that ST improves survival in patients with metastatic disease, particularly in lung and breast cancer populations.^8^^,^^10^^,^^11^ In our patient cohort, patients who received ST had median OS times over twice as long as the cohort who received no ST. It is also worth noting that the majority of our cohort was made up of lung and breast primary patients. We can conclude that our results support the conclusion that ST improves OS in patients with BrM, especially in lung and breast cancer patients, and suggest that ST should be initiated in this patient population regardless of relative timing with SRS. In our retrospective cohort, causality cannot be proved; larger and prospective studies are needed to investigate causality.

One rationale for initiating ST in close temporal proximity with SRS is to improve distant intracranial control. Patients with BrM are at risk of distant ICP and it is possible that prompt initiation of ST after SRS would lead to better intracranial control with less chance of reseeding the CNS.^1^^,^^5–7 ^^,^^12^ Indeed, it has been shown in certain populations that SRS delivered with ST has improved distant intracranial control compared with SRS alone. One recent series from our institution of 288 non-small cell lung cancer (NSCLC) and melanoma patients with BrM treated with SRS and immune checkpoint inhibitors (ICI) found that distant intracranial control was improved with the use of dual-ICI compared with SRS alone.^13^

However, the body of work that specifically evaluates the impact of the timing of ST with SRS on outcomes of patients with BrM is limited and primarily retrospective. Much of this data is conflicting on whether there are clear benefits from the timing of ST initiation. For example, the previously mentioned study from our institution did not show a benefit to overall survival or ICP from concurrent versus subsequent use of ICI in NSCLC and melanoma patients.^13^ But, there are several studies that show concurrent use or close temporal sequencing of ST with SRS increases intracranial control in some patient populations compared with longer intervals between therapies.^14–16^ We found that initiating ST within 3mo of SRS improved intracranial control on univariate analysis, compared with ST initiated after 3mo or not at all post-SRS. This may support the theory that, after SRS controls macroscopic tumor deposits, prompt initiation of ST is important to prevent reseeding of the brain from distant intracranial microscopic disease.

One difficulty in comparing these studies is that they often choose different time points of ST initiation as cutoffs for analysis. For example, our study examined ST initiated before and after 3mo from SRS, while the previously published study from our institution chose a 1mo time point.^13^ Additionally, some of these studies separate concurrent ST from ST administered shortly after completion of SRS, some do not make this distinction, and only some studies account for the pause in some ST that is typical around treatment with SRS.^12–16^ Randomized, prospective work is needed to determine the optimal interval between SRS and ST for specific agents and patient populations.

In our study, we found that, within breast cancer primary patients, ST within 3mo of SRS was associated with improved iPFS; notably, this was not true for the lung primary cohort. This supports several previously published series that have shown that HER2-directed therapy delivered concurrently with SRS shows improved control within the brain for breast primary patients.^17^^,^^18^ Further work is needed to investigate the effect of timing of ST and SRS on intracranial control in patients with metastatic breast cancer.

A topic of debate in the field has been whether administration of ST around the time of SRS increases the risk of developing radiation necrosis. We did not detect a differential rate of radiation necrosis based on receipt of ST following SRS in our cohort within primary cancer types. However, prior work has reported increased rates of radiation necrosis in patients given ST before or currently with SRS. A retrospective study from our institution in patients with HER2+ breast cancer BrM found that receipt of multiple HER2-directed ST agents within a 1mo window of SRS may increase the risk of developing radiation necrosis in these patients.^19^ Thus, given these disparate results, further work is needed to evaluate benefits and risks of initiating ST in patients with BrM.

There are several strengths of this study worth noting. Our cohort comprises a multi-institutional cohort of patients of a variety of primary types, which allows for a diverse and representative patient population. Additionally, our study is limited to patients with BrM alone; in other words, they could not have active ECD at the time of SRS, which would be a clear indication to start another therapy. Our study population allows us to specifically evaluate the effects of SRS and ST timing in patients with BrM alone.

There are several limitations of our work worth noting. Firstly, this is a retrospective study and as such subject to bias in terms of patient selection; we were not able to control which patients received ST and at what interval. For example, the ST >3mo cohort had a younger median age than the other two. This may suggest that these patients were otherwise healthier and preferred to delay the onset of ST and side effects, or it may imply that older patients are more likely to require more aggressive therapy, or no therapy at all due to poorer prognosis. Another possibly biased finding is that, within the lung primary patient cohort, those that did not receive ST had lower rates of post-SRS ECP than those who did receive ST. A possible explanation is that a portion of the patients who were started on ST were deemed by the treating providers to be at higher risk of developing further metastatic disease, compared with those who never received subsequent ST; then, the higher risk patients did go on to progress. Yet, another source of bias is that the majority of patients in the ST <3mo cohort developed BM within 1mo of primary diagnosis. This cohort may have inherently different disease biology and thus different courses of progression. We attempted to control for some of these factors using multivariate models; however, these confounding factors limit the interpretation and applicability of our findings.

Another limitation is that about 73% of this cohort had lung primaries and our results may be less applicable to other primary sites. Lung primary patients may be more likely to benefit from novel ST agents, such as immunotherapies and targeted agents, than other cancer types.

A third limitation is that we did not capture ST agent-specific information and were unable to delineate between different classes of systemic therapies. There are obvious and significant differences between ST classes and these differences no doubt account for some of the findings seen in our study. Further work is needed using specific agents, likely in specific populations of patients, to build upon our findings and further investigate the impact of ­timing between SRS and ST.

In conclusion, we found that in a population with brain-only metastatic disease, initiation of ST after SRS is associated with improved OS regardless of timing, and there was a suggestion of an improvement in distant intracranial control if ST was started within 3mo of SRS. The optimal interval between SRS and ST has yet to be determined and is likely influenced by a variety of patient and treatment factors. The complexity of the management of patients with BrM highlights the need for appropriate patients in this population to be referred for multidisciplinary discussion for the optimal sequencing of both local therapies, like SRS, and ST.

## Supplementary Material

vdaf214_Supplementary_Data

## Data Availability

The data underlying this article are available upon reasonable request and approval by institutional review board; details of data transfer pending approval.

## Lay Summary

Brain metastases are tumors that have spread to the brain from other parts of the body, and sometimes they are the only place the cancer is found. The authors of this study wanted to understand whether medicine that treats the whole body, called systemic therapy, helps people live longer after focused radiation to the brain. To do this, they looked at 342 people who had brain radiation and grouped them by whether they received this type of medicine within 3 months, after 3 months, or not at all. Their results showed that people who received systemic therapy after radiation lived longer than those who did not, no matter when the medicine was started.
