# Supplementary material for: Impact of systemic therapy after stereotactic radiosurgery in patients with limited brain-only metastasis
Source: Neurooncol Adv. 2025 Nov 20;7(1):vdaf214. doi: 10.1093/noajnl/vdaf214 (PMC12768501; doi:10.1093/noajnl/vdaf214)
Supplement: vdaf214_Supplementary_Data [file vdaf214_supplementary_data.docx]

**Supplementary Figure Captions**

Table 4: Multivariate Cox regression model evaluating predictors of extracranial progression free survival (ePFS).

Table 5: Chi-square analysis of extracranial progression (ECP) within breast primary patients.

Table 6: Chi-square analysis of extracranial progression (ECP) within lung primary patients.

**Supplementary Figures**

Table 4: Multivariate model of ePFS controlling for covariates

| **Parameter** |  | **p-value** | **Hazard Ratio** | **95% Hazard Ratio Confidence Limits** | |
| --- | --- | --- | --- | --- | --- |
| **Systemic therapy timing** | **No sys therapy** | 0.0634 | 1.703 | 1.016 | 2.855 |
|  | **Sys Therapy more 3 mo after local** |  | 1.010 | 0.651 | 1.566 |
| **Age at SRS** |  | 0.0249 | 0.982 | 0.966 | 0.998 |
| **Karnofsky Performance Status** | **<=80** | 0.1910 | 1.252 | 0.894 | 1.755 |
| **Metastatuc burden** | **5 or more metastases** | 0.9904 | 1.004 | 0.548 | 1.837 |
| **Primary site** | **Breast** | 0.1312 | 0.352 | 0.125 | 0.991 |
|  | **Lung** |  | 1.703 | 1.016 | 2.855 |
| **Systemic therapy prior to SRS** | **No** | 0.3111 | 1.010 | 0.651 | 1.566 |

Table 5: Chi-square analysis of ECP within breast primary patients

P = 0.17

| **ECP of breast primary patients** | | | | |
| --- | --- | --- | --- | --- |
| **ECP post SRS** | **Timing of Systemic Therapy** | | | |
|  | **Within 3 mo of SRS** | **3 mo after SRS** | **No sys therapy after SRS** | **Total** |
| **No (n, %)** | 3, 100.00 | 8, 50.00 | 15, 71.43 | 26 |
| **Yes (n, %)** | 0, 0.00 | 8, 50.00 | 6, 28.57 | 14 |
| **Total** | 3 | 16 | 21 | 40 |

Table 6: Chi-square analysis of ECP within lung primary patients

P<0.0001

| **ECP of breast primary patients** | | | | |
| --- | --- | --- | --- | --- |
| **ECP post SRS** | **Timing of Systemic Therapy** | | | |
|  | **Within 3 mo of SRS** | **3 mo after SRS** | **No sys therapy after SRS** | **Total** |
| **No (n, %)** | 26, 37.68 | 22, 36.07 | 72, 61.02 | 120 |
| **Yes (n, %)** | 43, 62.3 | 39, 63.9 | 46, 39.0 | 128 |
| **Total** | 69 | 61 | 118 | 248 |
